# Generative AI summarization tools and deep reading comprehension among Chinese university students: the mediating role of elaborative processing and the moderating role of reading motivation

**DOI:** 10.3389/fpsyg.2026.2002898

**Published:** 2026-10-16

**Authors:** Ruyu Bai

**Affiliations:** Executive Office, Changchun Finance College, Changchun, China

**Keywords:** AI summarization tools, deep reading comprehension, elaborative processing, generative AI, moderated mediation, reading motivation

## Abstract

Generative AI tools that condense texts into summaries have become a routine part of student reading routines, and educators increasingly worry that convenient summaries displace the effortful processing on which deep comprehension depends. This study drew on cognitive offloading theory and the levels-of-processing framework to test a moderated mediation model in which elaborative processing statistically links generative AI summarization tool use to deep reading comprehension, and reading motivation moderates the negative association between tool use and processing. Undergraduate students (*N* = 608; 53.6% female; mean age 19.9 years) from three universities in Changchun, China, completed a six-item scale of generative AI summarization tool use, an eight-item elaborative processing scale adapted from the deep-processing subscale of the R-SPQ-2F, a seven-item reading motivation measure adapted from the Motivation for Reading Questionnaire, and a researcher-developed deep reading comprehension test. Scale reliabilities were satisfactory (Cronbach alpha = 0.88, 0.87, and 0.86). Tool use correlated negatively with elaborative processing (*r* = −0.41) and comprehension (*r* = −0.24); elaborative processing correlated positively with comprehension (*r* = 0.59). PROCESS analyses (Model 7) with 5,000 bootstrap resamples showed that tool use negatively predicted elaborative processing (*b* = −0.310, *p* < 0.001) and that this association weakened as reading motivation rose (interaction *b* = 0.433, *p* < 0.001). Elaborative processing, in turn, predicted comprehension (*b* = 10.097, *p* < 0.001), whereas the residual direct path of tool use did not reach significance. The indirect effect of tool use on comprehension through elaborative processing was significant and negative at low (−6.142, 95% BC CI [−7.353, −5.091]) and average (−3.130, [−3.851, −2.497]) reading motivation but nonsignificant at high reading motivation (−0.117, [−0.876, 0.629]); the index of moderated mediation was 4.374 [3.428, 5.402]. These results are consistent with an account in which elaborative processing statistically links summary reliance to lower deep reading, with reading motivation associated with a weaker negative indirect association; given the cross-sectional design, the modeled paths represent associations rather than established causal effects. They suggest that motivation-oriented reading instruction should accompany, rather than follow, the integration of AI tools into academic reading.

## 1 Introduction

Large language models entered higher education with unusual speed. Within two academic years, generative AI moved from novelty to infrastructure in many institutions, prompting both enthusiasm about personalized support and unease about what students stop doing when a machine drafts, explains, and summarizes for them (Dwivedi et al., 2023; Holmes and Tuomi, 2022; Kasneci et al., 2023). Systematic reviews of AI in higher education document a research base that has grown faster than its evidence quality, with few studies tracing how specific AI practices reshape specific cognitive processes (Bond et al., 2024; Zawacki-Richter et al., 2019). Students themselves report pragmatic, task-focused adoption: tools that save time on assignments are welcomed, while institutional guidance lags behind actual use (Chan and Hu, 2023). Chinese campuses are no exception. Among Chinese university students, AI literacy predicts how confidently learners deploy these tools in language study (Zhu et al., 2026), and perceived usefulness shapes both engagement with generative AI and the risk of over-reliance on it (Liu and Liu, 2026).

Summarization is arguably the most consequential of these uses for reading. A summary tool collapses a text into its alleged essentials before a reader has engaged with the full passage, and recent work with Chinese mobile readers shows that reading AI-generated summaries in place of source texts changes trust calibration and comprehension outcomes in ways that depend on how the summary is used (Fan et al., 2026). On Chinese campuses the practice is ordinary rather than experimental: domestic assistants such as DeepSeek, Kimi, and Wenxin Yiyan offer one-tap condensation of articles, PDFs, and web pages, so a student who never installs a foreign chatbot still encounters machine summaries inside everyday study apps. Experimental evidence on chatbot assistance presents a mixed picture: a meta-analysis of controlled studies found that ChatGPT-based instruction improved learning performance overall, yet gains concentrated in tasks where the tool supported reasoning rather than replaced it (Deng et al., 2025). Reviewers of AI dialogue-system research warn that heavy reliance on conversational AI is associated with weaker performance on tasks demanding independent analysis and judgment (Zhai et al., 2024). What remains underspecified is the psychological route through which summary reliance helps or harms a durable academic capacity such as reading comprehension.

Empirical work on AI-assisted learning is beginning to map the conditions under which these tools help or harm. AI-supported personalized learning has been linked to higher-order thinking and creativity (Simsek et al., 2025), generative-AI-supported collaborative problem solving to better student performance (Tian et al., 2026), and the incorporation of generative AI into inquiry-based mathematics instruction to improved learning outcomes and motivation (Jia and Chiu, 2026). Generative AI has also been shown to promote pre-service teachers’ Technological Pedagogical Content Knowledge through a situational learning pathway (Yang et al., 2025), and AI-enhanced video-based situated learning environments can support teaching-design competence (Li et al., 2025). What these studies share is a focus on structured, instructional uses; unstructured reliance patterns such as habitual summary use remain much less examined.

A complementary line of work distinguishes use from reliance. Generative-AI use has been linked to learning engagement through approaches to learning and human-idea collaboration and cocreation (Wang and Guo, 2026), and students’ acceptance of AI in collaborative learning is shaped by social influence and attitudes (Wang et al., 2026a). Recent motivational models sharpen the distinction: the aMAZE model specifies approach and avoidance motivation in AI-assisted learning (Liu and Wang, 2024), and the EAGER framework differentiates engagement-driven from reliance-driven usage patterns (Wang et al., 2026b). Within this distinction, summary reliance represents a reliance-driven pattern whose correlates for reading have not been tested.

Two observations sharpen that question. Reading research has long distinguished literal extraction from the deeper processes of inference, integration, and evaluation that carry comprehension of expository text (Wolf and Barzillai, 2009), and international assessment frameworks build their reading scales on exactly these processes. A well-formed machine summary preserves most literal content: main claims, topic sequence, and conclusions survive the compression. What a summary cannot preserve is the reader-side work of building the situation model, tracking how paragraph four qualifies paragraph one, and deciding which claim deserves trust. If AI summaries erode comprehension, the erosion should therefore appear in deep reading specifically, through the processing activities a student no longer performs, rather than through confusion about stated facts. That expectation motivates the mediator examined here.

Cognitive offloading theory offers one such route. People routinely shift demanding cognitive operations onto external resources, and this shift is often adaptive in the moment while leaving internal representations weaker than practice would have built them (Risko and Gilbert, 2016). The classic demonstrations are direct: remembering that information is stored elsewhere reduces memory for the information itself (Sparrow et al., 2011), and even the act of saving a file improves later recall of what was saved less than continued internal rehearsal does (Storm and Stone, 2015). Distributed-cognition accounts frame modern devices as legitimate partners in thinking, but they also distinguish partnerships that extend internal processing from those that quietly substitute for it (Grinschgl and Neubauer, 2022). Generative AI intensifies the question, because language models offload not storage but the construction of meaning itself. Survey work linking frequent AI tool use to lower self-reported critical thinking (Gerlich, 2025), evidence that offloading erodes learning accountability in AI-mediated study (Liu et al., 2026), and Chinese data tying specific usage patterns to self-rated analytical abilities (Zhang and Yang, 2026; Zou et al., 2024) all point to the same concern: when the machine does the reading, who does the thinking?

Deep reading names the capacity at stake. In the account developed by reading researchers, deep reading goes beyond literal extraction to inference, integration across sentences and paragraphs, evaluation of claims, and connections to prior knowledge (Wolf and Barzillai, 2009). Two decades of medium-comparison research supply a cautionary base rate: meta-analytic evidence shows comprehension advantages for paper over screen reading that grow with time pressure and text complexity (Delgado et al., 2018), and systematic reviews converge on the same small but reliable screen penalty for expository text (Clinton, 2019; Mangen et al., 2013). Those findings concern print versus pixels. Summary tools change the object of engagement more radically, replacing the text with a compressed surrogate and inviting readers to inspect rather than construct its meaning.

The levels-of-processing framework explains why such replacement can matter. Retention and comprehension improve when learners engage material semantically rather than superficially, a principle established across verbal-learning experiments and still central to educational psychology (Craik and Lockhart, 1972; Craik and Tulving, 1975). In study-strategy research, the deep-processing subscale of the Revised Two-Factor Study Process Questionnaire captures the corresponding student orientation: relating ideas to prior knowledge, seeking examples, and transforming content rather than reproducing it (Biggs et al., 2001). We treat this orientation, labeled here elaborative processing, as the proximal cognitive activity that summary reliance may displace. A student who commands a fluent machine summary faces less pressure to build inferential links, test understanding, or restate ideas in personal terms, all hallmark activities of elaborative processing.

Reading motivation supplies the moderating condition. Motivation research shows that reading amount and breadth track motivation quality, with intrinsic reasons for reading predicting engagement and comprehension more strongly than extrinsic ones (Wigfield and Guthrie, 1997). Multidimensional work confirms that curiosity and involvement facets relate to reading behavior and competence in ways that outlook-based facets do not (Schiefele et al., 2012), consistent with self-determination theory’s account of intrinsic motivation as a resource that sustains effortful activity (Ryan and Deci, 2000). Among Chinese learners, reading motivation predicts comprehension of demanding classical texts (Lau, 2019), and strategy instruction that raises motivation also raises comprehension for Chinese secondary readers (Li et al., 2022). A motivated reader, we propose, has both a reason to accept the labor of elaboration and a reason to check the machine, treating summaries as scaffolds rather than substitutes. Reading motivation in this study is conceptualized as a primarily intrinsic orientation, following the intrinsic reading motivation tradition (Ryan and Deci, 2000; Schiefele et al., 2012): students read because reading itself is engaging and meaningful, not to obtain external outcomes. The motivational pathways identified in recent AI-learning models (Liu and Wang, 2024; Wang et al., 2026b) are therefore directly relevant to how such an orientation may condition the correlates of summary reliance. Reading motivation should therefore attenuate the negative link between summarization-tool reliance and elaborative processing, and with it the indirect cost to comprehension.

This study assembled these elements into a moderated mediation model estimated in a sample of 608 undergraduates from three universities in Changchun, China. Generative AI summarization tool use served as the predictor, elaborative processing as the mediator, deep reading comprehension as the outcome, and reading motivation as the first-stage moderator. Four hypotheses guided the analysis. First, summarization tool use would correlate negatively with deep reading comprehension. Second, elaborative processing would mediate this association, such that heavier tool use predicts less elaborative processing, which in turn predicts lower comprehension. Third, reading motivation would moderate the path from tool use to elaborative processing, buffering the negative association. Fourth, the indirect effect of tool use on comprehension through elaborative processing would itself vary with reading motivation, weakening from low to high motivation levels.

This design makes three contributions. First, it provides the first empirical test of how a specific and increasingly prevalent AI usage pattern, summary reliance, relates to deep reading comprehension among Chinese undergraduates, a population under-represented in the emerging evidence base on AI-assisted learning. Second, it jointly models the mediating pathway and a theoretically motivated moderator, asking not only whether but for whom summary reliance is associated with lower comprehension. Third, it distinguishes use from reliance, clarifying that the risk lies in a reliance-driven usage pattern rather than in any contact with machine-generated summaries.

## 2 Materials and methods

### 2.1 Participants

Undergraduates were recruited from three institutions in Changchun, Jilin Province, China: a finance college, a normal university, and a multidisciplinary university. Invitation letters went to instructors of general education courses, and whole classes volunteered during regular teaching hours; 13 intact classes participated (5, 5, and 3 across the three institutions; mean class size 46.8), and students had been administratively assigned to their class at enrolment rather than self-selecting into it. Of 645 students who received the materials, 37 were removed for failing an instructed attention item or for incomplete responses, leaving 608 valid cases (53.6% female; ages 17 to 24 years, M = 19.9, SD = 1.20). Year of study distribution was 198 first-year, 146 s-year, 151 third-year, and 113 fourth-year students, with majors spanning economics and management (*n* = 215), humanities and social sciences (*n* = 126), science and engineering (*n* = 125), arts and physical education (*n* = 79), and other fields (*n* = 63). Daily recreational reading outside coursework was below 30 min for 259 students and exceeded 2 hours for only 41. A sensitivity computation treating the 608 students as independent observations indicated that the final sample provided power above 0.85 for detecting population correlations as small as |r| = 0.13 and above 0.95 for the interaction and indirect effects at the magnitudes eventually observed. These are best-case values under simple random sampling; because students were nested within 13 intact classes, design-based sensitivity analyses with the class as the clustering unit were also conducted (see Data Analysis and the Sensitivity to class-level clustering section).

### 2.2 Measures

Generative AI summarization tool use. Six items were written for this study to capture reliance on generative AI summaries in academic reading, informed by documented usage patterns among Chinese students (Fan et al., 2026; Liu and Liu, 2026). An initial pool of nine items was reviewed by two educational psychology faculty members for content coverage and clarity and then piloted with 42 students from a class that did not join the main study; three items with low item-total correlations or ambiguous wording were dropped. Sample items are “When a course reading is long, I ask an AI tool to summarize it instead of reading the full text” and “I rely on AI-generated summaries to grasp the main points of assigned readings.” Responses used a five-point scale from 1 (strongly disagree) to 5 (strongly agree). One reverse-worded item was recoded before averaging. The full item wording is reported in Supplementary Table S1. Cronbach alpha was 0.88.

Elaborative processing. Eight items were adapted from the deep-processing subscale of the Revised Two-Factor Study Process Questionnaire (Biggs et al., 2001), reworded for reading contexts. Sample items are “While reading, I try to connect new ideas with what I already know” and “I put course readings into my own words to check my understanding.” Two reverse-worded items were recoded before averaging. The scale thus indexes a self-reported orientation toward elaborative engagement rather than processing observed within any single reading episode, a distinction revisited in the limitations. The full item wording is reported in Supplementary Table S1. Cronbach alpha was 0.87.

Reading motivation. Seven items were adapted from the intrinsic and curiosity facets of the Motivation for Reading Questionnaire (Wigfield and Guthrie, 1997). Sample items are “I read because reading is genuinely enjoyable” and “I like to get lost in a demanding text.” One reverse-worded item was recoded before averaging. The full item wording is reported in Supplementary Table S1. Cronbach alpha was 0.86.

Deep reading comprehension. A test developed for this study assessed comprehension of two expository passages of about 1,100 and 1,300 words on topics unlikely to be familiar to participants, drawn from university-level general-education materials. Two experienced university teachers reviewed all items for content validity and answerability, and the test was piloted with 42 students from the same population who did not join the main study, leading to minor wording revisions. Each passage was followed by ten open-ended and short-answer items scored 0 to 5 against rubrics emphasizing inference beyond literal statements, integration of information across paragraphs, and evaluation of author claims, yielding a total score from 0 to 100. A sample item presents two statements from different sections that point in opposite directions and asks which claim the author ultimately supports and why, with full credit requiring evidence from both sections. Two trained raters independently scored a random 20% of responses; agreement was high (intraclass correlation = 0.93), and remaining responses were scored by a single rater. The three questionnaire instruments were translated into Chinese and back-translated into English by two bilingual instructors following standard procedures (Brislin, 1970), with discrepancies resolved through discussion.

### 2.3 Procedure

Data collection took place across 4 weeks in regular class sessions. In each session, participants first completed the deep reading comprehension test, allowing 25 min for reading and answering, and then the questionnaire battery, allowing 15 min. Trained assistants, none of whom taught the participating classes, read standardized instructions, supervised completion, and collected materials on site. Participation was voluntary and anonymous; no course credit or payment was attached. The questionnaire included one instructed attention item (“Please select disagree for this item”) used solely for screening. The study was approved by the Institutional Review Board of Changchun Finance College and conducted in accordance with institutional requirements; all participants provided informed consent.

### 2.4 Data analysis

Data screening preceded all inference: no variable showed problematic distributional shape, and skewness and kurtosis values stayed within conventional bounds. Internal consistency was evaluated with Cronbach alpha (Cronbach, 1951). Because all questionnaire variables were self-reported, common method bias was examined with Harman single-factor analysis as a preliminary, though limited, diagnostic that does not control for method effects (Podsakoff et al., 2003, 2024). As a measurement check, a confirmatory factor analysis (CFA) of the 21 questionnaire items was fitted with a three-factor structure corresponding to the three scales, with fit evaluated by χ^2^, CFI, TLI, RMSEA, and SRMR. Hypotheses were tested with the PROCESS macro for SPSS (version 4.2), Model 7(Hayes, 2018), which estimates first-stage moderated mediation in a regression framework. Generative AI summarization tool use was the predictor, elaborative processing the mediator, deep reading comprehension the outcome, and reading motivation the moderator of the predictor-to-mediator path. Predictor and moderator were mean-centered, and conditional indirect effects were computed at reading motivation levels of one standard deviation below the mean, at the mean, and one standard deviation above the mean (Aiken and West, 1991). Indirect effects were tested with 5,000 bootstrap resamples and bias-corrected 95% confidence intervals, which avoid the normality assumption for product terms (Preacher and Hayes, 2008; Shrout and Bolger, 2002), and the index of moderated mediation evaluated whether the indirect effect varied systematically across moderator levels (Preacher et al., 2007). Analyses were run in SPSS 26.0, with effect sizes interpreted against conventional benchmarks (Cohen, 1988). Two-tailed tests with a significance criterion of 0.05 were used throughout, exact *p* values are reported except where they fall below 0.001, and no data were analyzed before the screening rules described above had been applied. Because the data are cross-sectional, mediation estimates can deviate from the longitudinal parameters of interest even under correct causal ordering (Maxwell et al., 2011); the modeled paths are therefore interpreted as associations consistent with the theoretical model rather than established causal effects. Because whole classes volunteered, students were nested within 13 intact classes. Class identifiers were not retained in the anonymized dataset, so class-adjusted standard errors cannot be computed directly. The design effect DEFF = 1 + (m − 1) × ICC (Kish, 1965) was therefore applied, with m = 46.8 (mean class size) and ICC varied from 0.01 to 0.10, the range commonly reported for cognitive and motivational outcomes in classroom studies (Hedges and Hedberg, 2007). For each path we report the range of design-adjusted t values and the ICC at which it would lose significance, together with bias-corrected bootstrap intervals for the indirect effects re-scaled by the design effect and the implied effective sample size. University-level intraclass correlations, estimable from the sampling-site indicators, were near zero (range −0.004 to 0.010).

## 3 Results

### 3.1 Preliminary analyses

Table 1 presents participant characteristics, and Table 2 reports descriptive statistics, reliabilities, and correlations. All three scales showed satisfactory internal consistency (alpha = 0.88 for summarization tool use, 0.87 for elaborative processing, and 0.86 for reading motivation). Skewness ranged from −0.39 to −0.07 and kurtosis from −0.45 to 0.22 across the four study variables, indicating no distributional concerns. As expected, summarization tool use correlated negatively with elaborative processing (*r* = −0.41), with deep reading comprehension (r = −0.24), and with reading motivation (*r* = −0.15). Elaborative processing correlated positively with reading motivation (*r* = 0.40) and strongly with comprehension (*r* = 0.59); reading motivation itself correlated with comprehension (*r* = 0.24). All reported correlations were significant at *p* < 0.001 with *N* = 608. Harman single-factor analysis extracted a first factor accounting for 31.15% of total variance, below the 40% threshold, suggesting that common method variance was not a dominant influence on the observed relationships (Podsakoff et al., 2003). The three-factor CFA supported the measurement structure: χ^2^(186) = 206.0, *p* = 0.150, CFI = 0.996, TLI = 0.996, RMSEA = 0.013, and SRMR = 0.026; all standardized loadings were significant, ranging from 0.65 to 0.75 (Supplementary Table S1), and composite reliability (CR = 0.86–0.88) and average variance extracted (AVE = 0.46–0.55) were acceptable. A single-factor model fit far worse (χ^2^(189) = 2372.5, CFI = 0.568; Δχ^2^(3) = 2166.5), further indicating that a single common method factor is unlikely to account for the findings.

**Table 1:** Participant characteristics (*N* = 608).

Category	*n*	%	Category	*n*	%
Gender			Year of study		
Female	326	53.6	First year	198	32.6
Male	282	46.4	Second year	146	24.0
Major field			Third year	151	24.8
Economics and management	215	35.4	Fourth year	113	18.6
Humanities and social sciences	126	20.7	Daily recreational reading		
Science and engineering	125	20.6	Below 30 min	259	42.6
Arts and physical education	79	13.0	30 to 60 min	208	34.2
Other	63	10.4	1 to 2 h	100	16.4
Sampling site			More than 2 h	41	6.7
Finance college	243	40.0			
Normal university	229	37.7			
Multidisciplinary university	136	22.4			

Age ranged from 17 to 24 years (M = 19.9, SD = 1.20). Sampling sites were three universities in Changchun, Jilin Province, China.

**Table 2:** Descriptive statistics, reliabilities, and correlations among study variables (*N* = 608).

Variable	M	SD	Skew	Kurt	Alpha	1	2	3	4
1. GSTU	3.11	0.78	−0.09	−0.33	0.88	–			
2. Elaborative processing	3.05	0.66	−0.39	0.22	0.87	−0.41	–		
3. Reading motivation	3.10	0.69	−0.13	−0.45	0.86	−0.15	0.40	–	
4. Deep reading comprehension	68.09	11.20	−0.07	0.09	–	−0.24	0.59	0.24	–

GSTU, generative AI summarization tool use. Alpha, Cronbach alpha; the comprehension score is a performance total with no alpha. All correlations are significant at *p* < 0.001.

### 3.2 Moderated mediation analysis

Table 3 reports the regression components. In the mediator model, summarization tool use negatively predicted elaborative processing (a1 = −0.310, SE = 0.027, *t* = −11.59, *p* < 0.001), reading motivation positively predicted elaborative processing (a2 = 0.306, SE = 0.030, *t* = 10.13, *p* < 0.001), and the tool use by reading motivation interaction was significant and positive (a3 = 0.433, SE = 0.038, *t* = 11.28, *p* < 0.001), indicating that the negative association of tool use with elaborative processing weakened as reading motivation rose. The interaction model explained 41.1% of the variance in elaborative processing. In the outcome model, elaborative processing strongly predicted deep reading comprehension (b1 = 10.097, SE = 0.613, *t* = 16.48, *p* < 0.001), while the residual direct path of tool use did not approach significance (c-prime = 0.065, *p* = 0.90); this model explained 34.9% of comprehension variance. The total association of tool use with comprehension, estimated without the mediator, was negative and significant (c = −3.444, SE = 0.569, *t* = −6.05, *p* < 0.001). Hypotheses 1 through 3 were supported.

**Table 3:** Regression results for the moderated mediation model.

Predictor	*b*	SE	*t*	*p*
Mediator model: elaborative processing				
GSTU (a1)	−0.310	0.027	−11.59	<0.001
Reading motivation (a2)	0.306	0.030	10.13	<0.001
GSTU x reading motivation (a3)	0.433	0.038	11.28	<0.001
Outcome model: deep reading comprehension				
GSTU (direct, c-prime)	0.065	0.519	0.13	0.900
Elaborative processing (b1)	10.097	0.613	16.48	<0.001
GSTU total effect on comprehension (c)	−3.444	0.569	−6.05	<0.001

*N* = 608. Unstandardized coefficients; GSTU and reading motivation mean-centered before forming the product term. The mediator model explained 41.1% of the variance in elaborative processing and the outcome model 34.9% of the variance in deep reading comprehension.

Simple slope analyses clarified the interaction. At one standard deviation below the mean of reading motivation, tool use predicted elaborative processing steeply and negatively (*b* = −0.608, SE = 0.038, *t* = −15.99, *p* < 0.001). At the mean, the slope remained negative (*b* = −0.310, SE = 0.027, *t* = −11.59, *p* < 0.001). At one standard deviation above the mean, the slope was indistinguishable from zero (*b* = −0.012, SE = 0.037, *t* = −0.31, *p* = 0.755). For students high in reading motivation, the association of summarization tool use with elaborative processing was not statistically detectable, whereas for students low in reading motivation each unit of tool use was associated with a substantial drop.

### 3.3 Conditional indirect effects

Table 4 reports bootstrap estimates for the conditional indirect effects and the index of moderated mediation. The indirect effect of summarization tool use on comprehension through elaborative processing was significant and negative at low reading motivation (effect = −6.142, boot SE = 0.580, 95% BC CI [−7.353, −5.091]) and at average reading motivation (effect = −3.130, boot SE = 0.347, 95% BC CI [−3.851, −2.497]), but not at high reading motivation (effect = −0.117, boot SE = 0.383, 95% BC CI [−0.876, 0.629]). The index of moderated mediation was significant (index = 4.374, boot SE = 0.505, 95% BC CI [3.428, 5.402]), confirming that the indirect association of tool use with comprehension varied with reading motivation. The residual direct effect was nonsignificant (0.065, 95% BC CI [−0.969, 1.057]), while the total effect remained negative (−3.444, 95% BC CI [−4.600, −2.254]). Hypothesis 4 was therefore supported: reading motivation moderated the mediated association between summarization tool use and comprehension.

**Table 4:** Conditional indirect effects and index of moderated mediation.

Effect	Estimate	Boot SE	95% BC CI
Indirect effect at low reading motivation (−1 SD)	−6.142	0.580	[−7.353, −5.091]
Indirect effect at average reading motivation (M)	−3.130	0.347	[−3.851, −2.497]
Indirect effect at high reading motivation (+1 SD)	−0.117	0.383	[−0.876, 0.629]
Index of moderated mediation	4.374	0.505	[3.428, 5.402]
Residual direct effect	0.065	0.519	[−0.969, 1.057]
Total effect	−3.444	0.608	[−4.600, −2.254]

*N* = 608. Bootstrapping with 5,000 resamples; bias-corrected confidence intervals. GSTU, generative AI summarization tool use. Conditional indirect effects computed at reading motivation levels of one standard deviation below the mean, the mean, and one standard deviation above the mean. The indirect effect is the product of the conditional effect of GSTU on elaborative processing and the effect of elaborative processing on deep reading comprehension. Two standard errors are reported for the total association: Table 3 gives the analytic (model-based) standard error, whereas this table reports the bootstrap estimate, defined as the standard deviation of the 5,000 bootstrap estimates; the two need not coincide.

### 3.4 Sensitivity to class-level clustering

As a second clustering check, the design-effect sensitivity analysis was applied with the class as the clustering unit. Design-adjusted t values remained significant for all four model paths across the full ICC range from 0.01 to 0.10 (*t* = 9.60 to 4.91 for the tool-use to elaborative-processing path, 9.34 to 4.78 for the interaction path, 13.65 to 6.98 for the elaborative-processing to comprehension path, and 5.01 to 2.56 for the total association); each path would lose significance only at ICC values well above the customary classroom range (0.74, 0.70, 1.52, and 0.18, respectively). At ICC = 0.10, the re-scaled bootstrap interval for the indirect effect remained below zero at low ([−8.83, −3.46]) and average ([−4.74, −1.52]) reading motivation and included zero at high motivation ([−1.89, 1.66]), mirroring the primary analysis, and the index of moderated mediation remained significant ([2.04, 6.71]). The effective sample size implied by the design effect was 185 at ICC = 0.05 and 109 at ICC = 0.10, still comfortably above what the observed effects require (observed *f*^2^ = 0.21–0.22 against a minimum detectable f^2^ of approximately 0.07 at ICC = 0.10). The substantive conclusions therefore hold under plausible levels of class-level clustering.

## 4 Discussion

This study asked how generative AI summarization tools relate to deep reading comprehension among Chinese university students, and what psychological resources moderate that relationship. In a sample of 608 undergraduates from three Changchun institutions, summarization tool use was negatively associated with comprehension, and this association traveled almost entirely through elaborative processing. The negative path from tool use to elaborative processing weakened as reading motivation increased, to the point that for highly motivated students the association was no longer statistically detectable, and the conditional indirect effect mirrored this pattern from low to high motivation.

The mediation result gives the offloading account a concrete location. Earlier work established that offloading storage degrades memory for the offloaded material (Sparrow et al., 2011; Storm and Stone, 2015) and framed the wider trade-off between internal effort and external support (Grinschgl and Neubauer, 2022; Risko and Gilbert, 2016). Our findings extend the logic from remembering to comprehending: what a summary tool invites students to offload is the construction of meaning, and the measurable consequence appears not in the residual direct path but in the quiet displacement of elaborative activity. Students who leaned on machine summaries reported less connecting, exemplifying, and restating of ideas, and this displacement, rather than any immediate confusion caused by the summaries themselves, statistically accounted for lower comprehension scores. The extension also carries a boundary worth stating: when a summary functions as a preview that students verify against the source, offloading part of the selection work can free attention for integration, so the association documented here reflects reliance rather than any contact with machine summaries. This pattern is consistent with motivational models that distinguish engagement-driven from reliance-driven AI use (Liu and Wang, 2024; Wang et al., 2026b). The pattern aligns with survey evidence linking heavy AI use to weaker analytical self-ratings among Chinese students (Zhang and Yang, 2026; Zou et al., 2024) and with the accountability erosion observed when generative AI absorbs study labor (Liu et al., 2026).

The moderation result identifies who is most exposed. Reading motivation, long tied to reading amount and comprehension quality (Schiefele et al., 2012; Wigfield and Guthrie, 1997), operated here as a buffer on the first stage of the mediated pathway. Two readings of this buffer deserve attention, and they are not mutually exclusive. First, motivation supplies the willingness to spend effort: self-determination theory holds that intrinsently valued activity is defended rather than abandoned when shortcuts appear (Ryan and Deci, 2000), and a reader who enjoys demanding texts has reasons to open the original passage even when a summary sits one tap away. Second, motivated readers may use tools differently rather than less, checking machine output against the source, a usage pattern that the trust-calibration work on AI summaries treats as productive (Fan et al., 2026). Chinese classroom evidence that motivation-oriented instruction raises comprehension for secondary readers (Lau, 2019; Li et al., 2022) suggests this buffer is educatable, not fixed.

The Chinese context of the study deserves one further note. The tools our items describe are woven into the study apps that Chinese students already use for search, note-taking, and document reading, which lowers the friction of summary reliance below that observed in settings where students must deliberately open a chatbot. Chinese universities have also integrated AI literacy into information literacy instruction at different speeds, and perceived usefulness rather than regulation tends to drive adoption among Chinese undergraduates (Liu and Liu, 2026; Zhu et al., 2026). Against that backdrop, our finding that a motivational resource, not a technical skill, buffered the comprehension cost carries a specific implication for Chinese institutions: AI literacy modules that emphasize verification routines may work best when paired with reading programs that give students texts worth wanting to read.

For policy and pedagogy, the findings suggest a tentative middle course between prohibition and laissez-faire, to be tested in instructional intervention studies. Because the association of tool use with comprehension traveled through elaborative processing, instruction that makes elaboration visible and rewarded could plausibly weaken it: summarization assignments that require students to verify machine summaries against source texts, justify edits, or reconstruct the argument from a summary are direct applications. Because reading motivation buffered the pathway, motivation-oriented practices, choice of reading material, curiosity-driven topic selection, and low-stakes discussion of enjoyable texts, belong in the same curriculum that adopts AI tools. A workable course sequence follows from the two principles together: students first read a short source text and record their own summary, then obtain the machine summary, then mark every place where the two versions diverge and defend one of them with evidence from the original passage. Such a cycle converts the tool from a substitute into an object of elaboration itself. The meta-analytic finding that ChatGPT assistance helps most when it supports rather than replaces reasoning (Deng et al., 2025) supports the same division of labor. Faculty development and library instruction that treat summary tools as objects of critical inspection rather than invisible infrastructure would enact it. Whether these adjustments change reading outcomes remains an instructional hypothesis that the present correlational design did not test.

Several limitations qualify these conclusions. The design was cross-sectional and correlational, so the directions modeled here, from tool use through processing to comprehension, represent theoretical commitments rather than demonstrated causation, and cross-sectional mediation estimates can deviate from the longitudinal parameters of interest even under correct causal ordering (Maxwell et al., 2011); students with weaker comprehension may gravitate toward summaries in the first place. All questionnaire measures were self-reported at one sitting; although Harman single-factor variance stayed below threshold, common method variance cannot be excluded, and the comprehension test, while performance-based, was researcher-developed and unreplicated beyond double-scoring. The tool use measure asked about summarization specifically but not about how students actually deployed summaries, so verification behaviors remain unobserved. The elaborative processing measure likewise captures a habitual orientation, so the modeled displacement of processing activity is inferred from self-reports rather than traced within specific reading episodes. Although the three-factor structure of the adapted scales was supported in this sample, measurement invariance across gender, year, or institution subgroups is untested. Students were nested within 13 intact classes whose identifiers were not retained, so class-level clustering could be addressed only through design-effect sensitivity analyses rather than direct modeling. The sample came from three institutions in one Chinese city with a commerce-heavy major distribution, limiting generalization to other regions, academic cultures, and to readers outside the university years. Future work should combine log data on actual tool use with longitudinal comprehension measurement, examine second-stage moderation by task demands such as text difficulty or accountability, and test the instructional designs implied above under controlled conditions.

Within those limits, the study offers a tested statistical pathway rather than a warning label. Elaborative processing carried the association between generative AI summarization tool use and deep reading comprehension among Chinese university students, and reading motivation moderated the pathway so strongly that motivated readers showed no measurable indirect cost. The reading crisis narrative that sometimes accompanies discussions of AI in education is not what these data support. They support a narrower and more actionable claim: the negative association of summary reliance with comprehension is concentrated among readers least inclined to elaborate and is not detectable where reading motivation is high, which identifies elaboration habits and reading motivation as candidate targets for intervention research.

## 5 Conclusion

Among 608 Chinese undergraduates, reliance on generative AI summarization tools was associated with lower deep reading comprehension through reduced elaborative processing, and reading motivation moderated this mediated pathway, rendering the indirect effect nonsignificant at high motivation. The results point to displaced elaboration, rather than the summaries themselves, as the locus of the association, and to reading motivation as a protective resource. Reading instruction that builds elaborative habits and motivation alongside, not after, the adoption of AI tools offers one practical route forward, pending confirmation in longitudinal and experimental designs.

## Data Availability

The raw data supporting the conclusions of this article will be made available by the authors, without undue reservation.
