# Supplementary material for: Generative AI summarization tools and deep reading comprehension among Chinese university students: the mediating role of elaborative processing and the moderating role of reading motivation
Source: Front Psychol. 2026 Oct 16;17:2002898. doi: 10.3389/fpsyg.2026.2002898 (PMC13719582; doi:10.3389/fpsyg.2026.2002898)
Supplement: Supplementary file 1 [file Presentation_1.pdf]

Supplementary Table S1

Item wording, standardized factor loadings, and reliability indices for the study measures

All questionnaire items used a five-point response scale from 1 (strongly disagree) to 5 (strongly agree). Items flagged R were reverse-scored (6 − x) before analysis. The deep reading comprehension test (performance-based, 0–100) is described in the Measures section of the main text.

Scale	Item	Wording	Reverse-scored	λ
Generative AI summarization tool use (GSTU)	X1	When a course reading is long, I ask an AI tool to summarize it instead of reading the full text.	No	0.744
	X2	I rely on AI-generated summaries to grasp the main points of assigned readings.	No	0.732
	X3	Before class, I usually read the AI summary rather than the assigned text itself.	No	0.747
	X4	When an AI summary is available, I see little need to open the original reading.	No	0.754
	X5	I feel confident answering questions about a reading after reading only its AI summary.	No	0.744
	X6	I make sure to read the full original text even when I have an AI summary of it.	Yes	0.728
Elaborative processing	M1	While reading, I try to connect new ideas with what I already know.	No	0.670
	M2	I put course readings into my own words to check my understanding.	No	0.676
	M3	When I finish a reading, I rarely think about how its ideas relate to other topics.	Yes	0.729
	M4	I ask myself questions about the text and try to answer them as I read.	No	0.648
	M5	I compare claims made in one reading with those in other readings or lectures.	No	0.670
	M6	I usually read through assigned texts once without stopping to reflect on them.	Yes	0.648
	M7	I try to work out real-world examples of the ideas presented in a reading.	No	0.673
	M8	After reading, I mentally reconstruct the author's argument from beginning to end.	No	0.684
Reading motivation	W1	I read because reading is genuinely enjoyable.	No	0.702
	W2	I like to get lost in a demanding text.	No	0.687
	W3	I look forward to courses that assign challenging readings.	No	0.674
	W4	I read only what I have to read to get by in my courses.	Yes	0.667
	W5	Finishing a difficult text gives me a sense of accomplishment.	No	0.709
	W6	I often read beyond the assigned portions of a text because I want to know more.	No	0.695
	W7	I would rather spend free time reading than doing most other activities.	No	0.652

Note. λ = standardized factor loading from the three-factor confirmatory factor analysis (N = 608): $\chi^2(186)$ = 206.0, p = .150, CFI = 0.996, TLI = 0.996, RMSEA = 0.013, SRMR = 0.026. Reliability by scale — GSTU: α =

0.88, CR = 0.88, AVE = 0.550; elaborative processing: α = 0.87, CR = 0.87, AVE = 0.456; reading motivation: α = 0.86, CR = 0.86, AVE = 0.468. GSTU, generative AI summarization tool use.
